# Supplementary material for: Heterogeneity in tumor chromatin-doxorubicin binding revealed by in vivo fluorescence lifetime imaging confocal endomicroscopy
Source: Nat Commun. 2018 Jul 9;9:2662. doi: 10.1038/s41467-018-04820-6 (PMC6037736; doi:10.1038/s41467-018-04820-6)
Supplement: Supplementary file 1 — Supplementary Information [file 41467_2018_4820_MOESM1_ESM.pdf]

Supplementary Information

Sparks et al., *"Heterogeneity in tumor chromatin-doxorubicin binding revealed by in vivo fluorescence lifetime imaging confocal endomicroscopy"*

Supplementary Note 1 .... **2**

In vitro study to quantify the contribution of doxorubicin emission in the confocal endomicroscope detection channel and its impact on the measured EGFP fluorescence lifetime 2

Supplementary Note 2 .... **5**

Potential sources of error in the fluorescence lifetime analysis of H1-EGFP-doxorubicin FRET 5

Supplementary Figure 1 .... **7**

Comparison of GFP and doxorubicin emission spectra 7

Supplementary Figure 2 .... **8**

Fluorescence image from H1-GFP labelled IGROV-1 cells using multiphoton LSM 8

Supplementary Figure 3 .... **9**

Determination of relative contribution of direct doxorubicin excitation to change in fluorescence lifetime 9

Supplementary Figure 4 .... **11**

Minimal direct doxorubicin excitation in vivo and lack of photobleaching 11

Supplementary Figure 5 .... **13**

Longitudinal imaging of mice at 45, 90 and 180 minutes following IP delivery of doxorubicin. 13

Supplementary Figure 6 .... **14**

H1-EGFP lifetime analysis for every field of view for 31 mice including all drug delivery routes and times 14

Supplementary Figure 7 .... **15**

Calculated doxorubicin dose for every field of view for 31 mice including all drug delivery routes and times 15

Supplementary Figure 8 .... **16**

Histology of control and doxorubicin treated tumors 16

Supplementary Table 1 .... **17**

Measured fluorescence lifetime components 17

Supplementary Table 2 .... **18**

Summary of in vivo experiments 18

Supplementary References **19**

Supplementary Note 1

In vitro study to quantify the contribution of doxorubicin emission in the confocal endomicroscope detection channel and its impact on the measured EGFP fluorescence lifetime

The confocal endomicroscope (CEM) employs 488 nm excitation and a 520-550 nm detection band to detect GFP fluorescence. However, doxorubicin (DOX) is also excited weakly at 488 nm and its emission spectrum bleeds through into the CEM detection window. Supplementary Figure 1 shows the normalized emission spectra measured using the LSM of H1-EGFP with 488 nm excitation and DOX with 488 nm excitation together with the transmission profiles of the emission filters used for multiphoton LSM and CEM FLIM measurements. To quantify the relative contributions of GFP and doxorubicin to the fluorescence lifetimes measured with the CEM, we undertook an in vitro study using IGROV-1 cells expressing H1-EGFP.

First, we used the LSM system with two-photon excitation at 900 nm and the 465-495 nm band-pass emission filter to measure the change in H1-EGFP fluorescence lifetime upon treatment with doxorubicin due only to FRET upon doxorubicin binding to chromatin. Globally fitting a double exponential model to this FLIM dataset yielded a χ^2 goodness-of-fit parameter of less than 1.1 with 2330 ps (72%) and 770 ps (28%) for the H1-EGFP non-FRETing and FRETing lifetime components respectively.

To understand how the DOX fluorescence could contribute to fluorescence decay profiles measured in the CEM detection channel, we used the CEM system with 488 nm excitation and detection over 520-550 nm to measure the fluorescence decay profile of DOX within wild type IGROV-1 cell nuclei in vitro. We globally fitted to a double exponential decay model and obtained lifetime components of 1590 ps (35%) and 390 ps (65%) respectively with a χ^2 of less than 1.2.

In order to quantify the relative contributions of H1-EGFP and DOX measured with the CEM, we measured the emission spectrum (490–630 nm) of IGROV-1 cells expressing H1-GFP after treatment for 3 hours with 9 µM doxorubicin. This measurement was performed using 488 nm excitation with the emission being analyzed using the diffraction grating-based spectral imaging capability of a Zeiss LSM780 microscope and is represented by the blue curve of Supplementary Figure 2b. We also measured the emission spectra of IGROV-1 cells expressing H1-EGFP with no DOX treatment (yellow curve) and of wild-type IGROV-1 cells treated with 9 µM DOX for 3 hours (pink curve).

The combined fluorescence signal expected in the CEM detection band (520-550 nm), S_{CEM}, depends on the concentrations and the relative brightness of the two fluorophores, i.e.

$$S_{\mathrm{CEM}} = [\text{H1-EGFP in IGROV-1 nuclei}]s_{\mathrm{EGFP}} + [\mathrm{DOX}]s_{\mathrm{DOX}}$$

where [H1-EGFP in IGROV-1 nuclei] and [DOX] are the concentration of EGFP and DOX in IGROV-1 nuclei respectively and s_{EGFP} and s_{DOX} are the fluorescence signal per unit concentration of EGFP and DOX respectively. We used linear unmixing to estimate the ratio R of EGFP to DOX fluorescence in the CEM detection channel and found it to be 9.4 for 3 hours treatment with 9 µM DOX, where

$$R = \frac{[\text{H1-EGFP in IGROV-1 nuclei}]\, s_{\text{EGFP}}}{[9\ \mu\text{M}]\, s_{\text{DOX}}} = \frac{\text{Median measured EGFP IGROV-1 signal}}{\text{Median measured DOX IGROV-1 signal for 9 μM DOX}}$$

$$= \frac{S_{\text{EGFP IGROV-1}}}{S_{\text{DOX@9μM IGROV-1}}}.$$

As indicated in the equation above, *R* was calculated based on the cell-wise median EGFP and DOX nuclear fluorescence signals $S_{\text{GFP IGROV-1}}$ and $S_{\text{DOX@9μM}}$ respectively obtained from the nuclei image, see Supplementary Figure 2a.

Assuming the fluorescence signal from doxorubicin scales linearly with doxorubicin concentration, the expected signal S_{DOX} due to doxorubicin can be estimated for an arbitrary doxorubicin concentration by the equation

$$S_{\text{DOX IGROV-1}} = \frac{[\text{DOX}]}{[9\ \mu\text{M}]} \cdot S_{\text{DOX@9μM IGROV-1}} = \frac{[\text{DOX}]}{[9\ \mu\text{M}]} \frac{S_{\text{EGFP IGROV-1}}}{R}.$$

The CEM fluorescence decay that we expect to measure can be expressed as a linear sum of the H1-EGFP and DOX fluorescence decays detected in the 520-550 nm emission window. The total fluorescence signal as a function of time t after excitation for an infinitely narrow pulse of excitation light can therefore be described by a multi-exponential decay of four components: 2 for H1-GFP and 2 for doxorubicin

$$S_{\text{CEM}}(t) = S_{\text{EGFP IGROV-1}} \left\{ \frac{1}{\beta_{G1}\tau_{G1} + (1-\beta_1)\cdot\tau_{G2}} \cdot \left[\beta_{G1} \mathrm{e}^{-\frac{t}{\tau_{G1}}} + (1-\beta_{G1}) \cdot \mathrm{e}^{-\frac{t}{\tau_{G2}}} \right] + \frac{[\text{DOX}]/([9\ \mu\text{M}]\cdot R)}{\beta_{D1}\cdot\tau_{D1} + (1-\beta_{D1})\cdot\tau_{D2}} \cdot \left[\beta_{D1}\cdot \mathrm{e}^{-\frac{t}{\tau_{D1}}} + (1-\beta_D)\cdot \mathrm{e}^{-\frac{t}{\tau_{D2}}} \right] \right\}$$

and

$$\int_{-\infty}^{\infty} S_{\text{CEM}}(t)\mathrm{d}t = S_{\text{EGFP IGROV-1}}(1 + [\text{DOX}]/([9\ \mu\text{M}]\cdot R)).$$

Here τ_{G1} and τ_{G2} represent the long and short lifetime components of H1-EGFP; τ_{D1} and τ_{D2} represent the long and short lifetime components of DOX; β_{G1} represents the long lifetime fraction of GFP and β_{D1} represents the long lifetime fraction of DOX. The quantity $S_{\text{EGFP IGROV-1}}$ represents the total fluorescence signal from H1-GFP for the expression level achieved in our stable cell line.

The values of τ_{G1} were determined to be 2330 ps for multiphoton measurements and 2460 ps for CEM measurements. The value used for τ_{G2} was taken to be 770 ps, as determined with the multiphoton LSM in vitro measurements of IGROV-1 cells expressing GFP-H1 using a 465-495 nm emission filter to exclude the doxorubicin signal. It was not possible to measure a DOX-cross-talk-free value of τ_{G2} with the CEM. As discussed above, τ_{D1} and τ_{D2} were determined to be 1590 ps and 390 ps respectively from CEM in vitro measurements.

Using this approach to determine the relative fractions of the H1-EGFP non-FRETing and FRETing components and DOX bleed through contributions at each DOX concentration, Supplementary Figure 3a plots how the relative amounts of these contributions to the CEM signal vary as a function of DOX

concentration. The fraction of FRETing H1-EGFP component increases steeply for doxorubicin concentrations up to 1 µM and more gradually thereafter. The decreasing slope of the EGFP short component as a function of DOX concentration (blue curve in Supplementary Figure 3a) may reflect saturation of DOX binding sites.

To determine the intracellular DOX concentration from in vivo CEM measurements that may be compromised by the DOX bleed through, we compared the dose response curves for the in vitro measurements obtained with the multiphoton LSM and CEM instruments. The multiphoton LSM data was fitted to a double exponential decay model with τ_{G1} and τ_{G2} fixed to 2330 ps and 770 ps respectively and the CEM data was fitted to a double exponential decay model with τ_{G1} and τ_{G2} fixed to 2460 ps and 770 ps respectively. As shown in Supplementary Figure 3c that plots the % population fraction of the non-FRETing components, the curves are similar up to 1 µM DOX. Above this concentration, the CEM appears to underestimate the non-FRETing H1-EGFP contribution because of the DOX bleed through.

We used the in vitro data presented in Supplementary Figure 3c as a calibration to correct the in vivo CEM measurements. The in vivo CEM FLIM data was first fitted to a double exponential decay model with the non-FRETing and FRET component lifetimes fixed to 2376 ps and 770 ps respectively – noting that the non-FRETing H1-EGFP lifetime is that measured in vivo for control mice without doxorubicin treatment. The β_{G1} value obtained from this in vivo data can then be corrected according to a look-up table obtained by interpolating the data plotted in Supplementary Figure 3c. This corrected value of β_{G1} can then be used to estimate the intracellular DOX concentration that would have been measured in the absence of bleed through of the DOX fluorescence in the CEM spectral detection window.

To illustrate that the CEM measured DOX response data is not dominated by the DOX bleed through, fluorescence decay profiles were simulated for the 520-550 nm CEM detection band using the expressions above for doxorubicin concentrations in the range of 0 – 20 µM, as used in vitro and expected in vivo. Using the lifetime components for the in vitro CEM measurements (i.e. 2460 ps as measured with the CEM for non-FRETing H1-EGFP and 770 ps for FRETing H1-EGFP as measured without doxorubicin bleed through with the multiphoton LSM), Supplementary Figure 3d shows the simulated % of non-FRETing H1-EGFP in the detected CEM signal expected for the situation where there is no quenching of the H1-EGFP fluorescence but a contribution from DOX bleed-through (red curve) and where there is a contribution from quenched H1-EGFP fluorescence as well as the DOX contribution (blue curve). Also shown is the measured CEM signal as a function of DOX concentration (magenta curve). These results indicate that directly excited doxorubicin bleed through provides only a minor contribution to the CEM fluorescence lifetime measurements and the observed increase in the population fraction of FRETing H1-EGFP is the major factor in the reduction of fluorescence lifetime response to doxorubicin measured with the CEM.

Supplementary Note 2

Potential sources of error in the fluorescence lifetime analysis of H1-EGFP-doxorubicin FRET

To estimate the intracellular DOX concentrations, we have fitted the H1-EGFP emission decay profiles to a double exponential decay model that assumes a single FRET efficiency, i.e. that there is a longer lifetime fluorescence decay component representing the non-FRETing H1-EGFP emission and a single shorter lifetime component representing the FRETing H1-EGFP emission. This approach enables us to obtain reasonable decay χ^2 goodness of fit values with only a single adjustable fit parameter per pixel. When DOX binds to DNA, however, it can have a range of distances relative to the nearest EGFP-tagged H1 protein and there can be a range of angles between the DOX and EGFP fluorophore dipole orientations, so there will be a distribution of Förster resonant energy transfer efficiencies between EGFP and DOX. However, similar considerations apply to many FRET measurements of populations of fluorophores and it is common practice to analyse FLIM FRET data in this way. A further consideration is that the EGFP fluorophore is effectively static during its fluorescence decay and so the conventional assumption that FRET measurements average over a rapidly varying set of random orientations between the donor and acceptor fluorophore dipoles is not valid. This consideration is relevant to all FRET data measured with fluorescent proteins and means that the donor fluorescence will not be fully represented by a discrete exponential decay model [1]. Here, however, because we apply the same FLIM/FRET analysis to both the in vitro calibration data and the in vivo data, any bias due to the fitting model used should cancel as we apply our look-up table correction to obtain the estimated equivalent in vitro DOX concentrations.

Our simulation above shows that the DOX fluorescence accounts for ~1% of the total fluorescence signal for an in vitro DOX concentration of 1 µM (Supplementary Figure 3b). This is supported by direct comparison of in vitro LSM and CEM data, which shows good agreement in the measured non-FRETing H1-EGFP fractions up to a DOX contribution of ~2 µM (Supplementary Figure 3c). Above this DOX concentration, the effect of DOX fluorescence on the measured decay becomes more significant and we use the correction procedure based on a look-up table derived from Supplementary Figure 3c to convert the measured in vivo fraction of FRETing H1-GFP to the equivalent in vitro DOX concentration. While this correction is valid provided that the bleed-through contribution of DOX to the detected fluorescence signal is the same in vivo and in vitro for a given DOX concentration, there are a number of factors that may affect the validity of this assumption. First, DOX fluorescence intensity has been previously shown to be sensitive to its bound state [2,3], which may be different in vivo compared to in vitro. To reduce the potential effect of this on our analysis, we have used nuclear image segmentation to exclude potential contributions from unbound cytoplasmic DOX. We also note that DOX has previously been shown to have an intracellular degradation product with a longer fluorescence lifetime [4]. Again, any effect from this will be greatly reduced by the nuclear image segmentation applied because this degradation product was observed to accumulate in the cytoplasm. A further issue is that the fluorescence lifetime of DOX has been shown to be sensitive to the degree of chromatin condensation [5], which also may be different in vivo compared to in vitro. However, as the range of change in DOX lifetime with cell state varies by less than a factor of 2 [5] and, given that the unwanted DOX signal in our CEM data is a small fraction of the total fluorescence, we believe that this effect will not greatly perturb our results, particularly for lower DOX concentrations.

We note that future instrumentation incorporating improved fiber-optic bundles with different optical properties may be able to utilize shorter wavelength detection bands in the CEM that can eliminate the unwanted bleed-through contribution from DOX fluorescence.

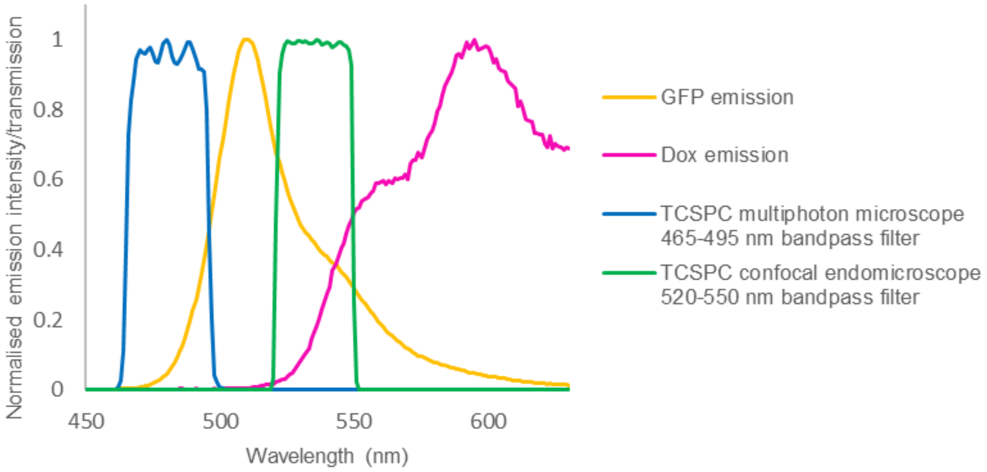

Supplementary Figure 1

Comparison of GFP and doxorubicin emission spectra

Transmission profiles of emission filters used with multiphoton LSM and CEM are shown overlaid on normalized emission spectra of GFP and doxorubicin. For the multiphoton LSM, the emission filter (blue curve) excluded doxorubicin emission. The longer 520-550 nm band-pass emission filter for the CEM (green curve) does not exclude all doxorubicin fluorescence. This filter was required to block Raman scattering of the excitation light and background fluorescence from the fiber-optic bundle.

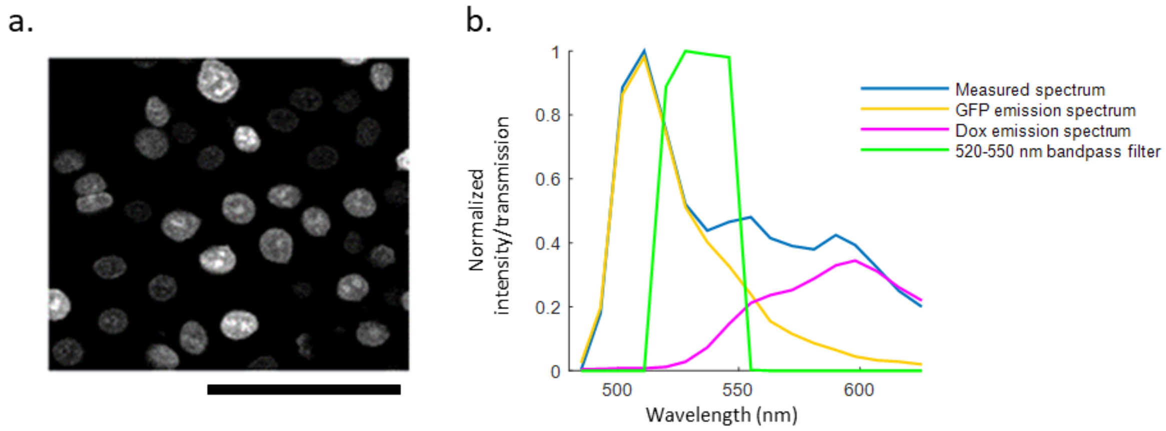

Supplementary Figure 2

Fluorescence image from H1-GFP labelled IGROV-1 cells using multiphoton LSM

(**a**) fluorescence intensity image, detected across 490-630 nm, of IGROV-1 histone H1-GFP cells treated with 9 µM doxorubicin is shown (scale bar is 70 µm). (**b**) Plot of emission spectra obtained from 33 cell nuclei in the image data presented in (**a**). Blue line shows the measured signal comprising emission from H1 GFP and doxorubicin. The yellow and magenta curves show the weighted mixture of the pure spectra for H1-GFP and doxorubicin emission which were measured beforehand from IGROV-1 histone H1-GFP cells not treated with doxorubicin and unlabeled IGROV-1 cells treated with 9 µM of doxorubicin for 3 hours respectively. The emission filter used in the CEM (green curve), shows the bleed through contribution of doxorubicin to the total signal detected using the CEM's 520 550 nm band-pass emission filter.

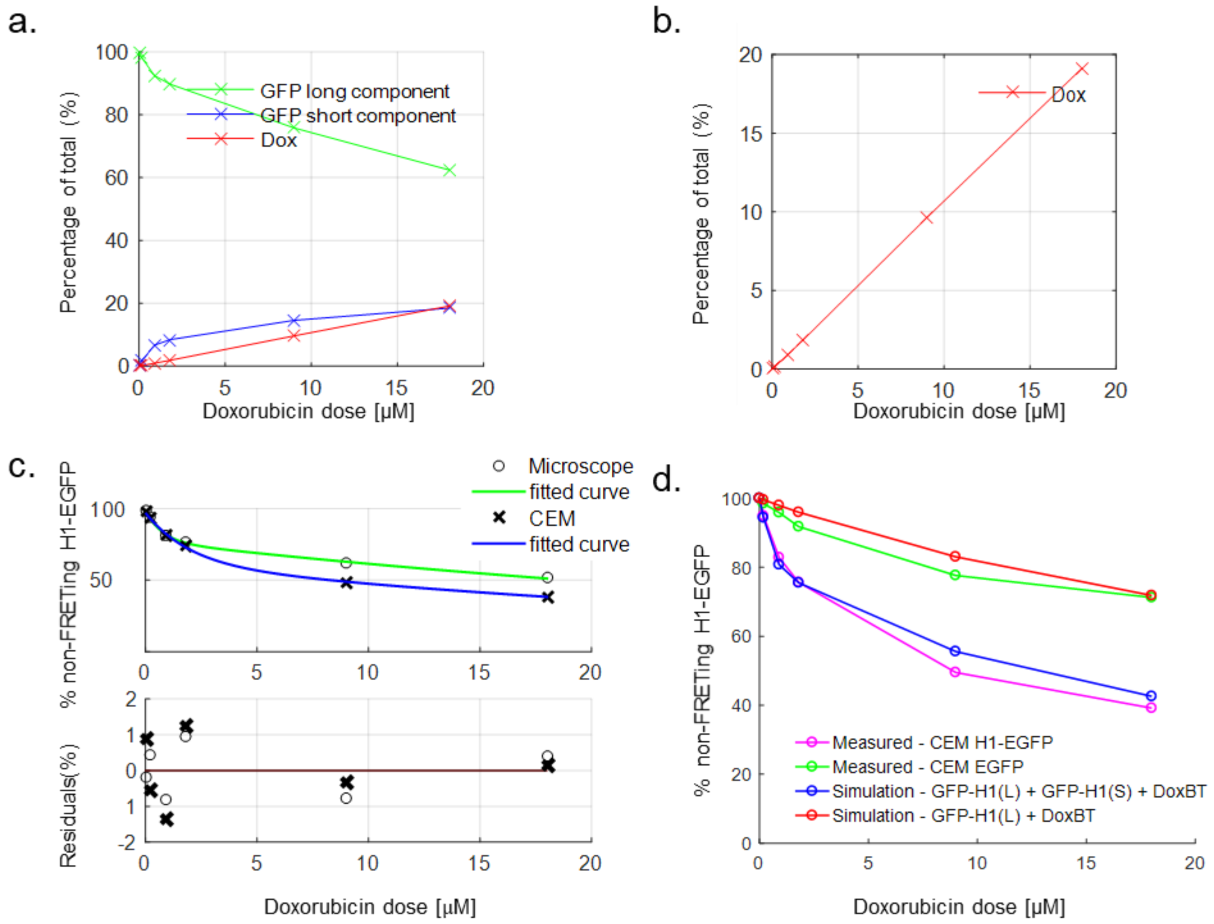

Supplementary Figure 3

Determination of relative contribution of direct doxorubicin excitation to change in fluorescence lifetime

(**a**) Simulations of relative amounts of fluorescence from the non-FRETing and FRETing H1-GFP (i.e. long and short H1-EGFP components) and doxorubicin bleed through in the CEM detection spectral window. The simulation is based on a 4-component fluorescence decay model as discussed in the Supplementary Note 1. (**b**) Doxorubicin contribution is plotted as a percentage of total signal for increasing doxorubicin concentrations. (**c**) Dose response curves obtained in vitro from measurements with the multiphoton LSM (circles) and CEM (crosses). These points were obtained by fitting the decay data to a double exponential decay model where τ_{G1} and τ_{G2} were fixed to 2330 ps and 770 ps respectively for the multiphoton LSM data and 2460 ps and 770 ps respectively for the CEM data. The vertical axis shows estimated percentage of non-FRETing H1-GFP fluorophores (β_G) for different doxorubicin doses for each instrument. The blue and green curves are double exponential fits to the CEM and LSM points respectively. The blue curve provides the calibration (look-up function) for the CEM – enabling us to estimate the equivalent intracellular doxorubicin dose (accounting for DOX bleed through) from a CEM measurement of β_{G1}. The lower plot shows the residual differences between the experimental LSM and CEM data and their respective double exponential fit. (**d**) Shows a comparison of our simulations of the fluorescence signal in the CEM detection channel with the experimental in vitro data (magenta curve) as a function of

doxorubicin dose. The red curve simulates the expected signal if there was no quenching of H1-EGFP but just the bleed through of doxorubicin in the CEM detection band (DoxBT). The blue curve simulates the expected signal including the contributions from FRETing H1-GFP fluorophores and the doxorubicin bleed through. In all cases the simulated and measured fluorescence lifetime data were fitted in *FLIMfit* to a double exponential decay model. The crosses and circles in all plots represent data points.

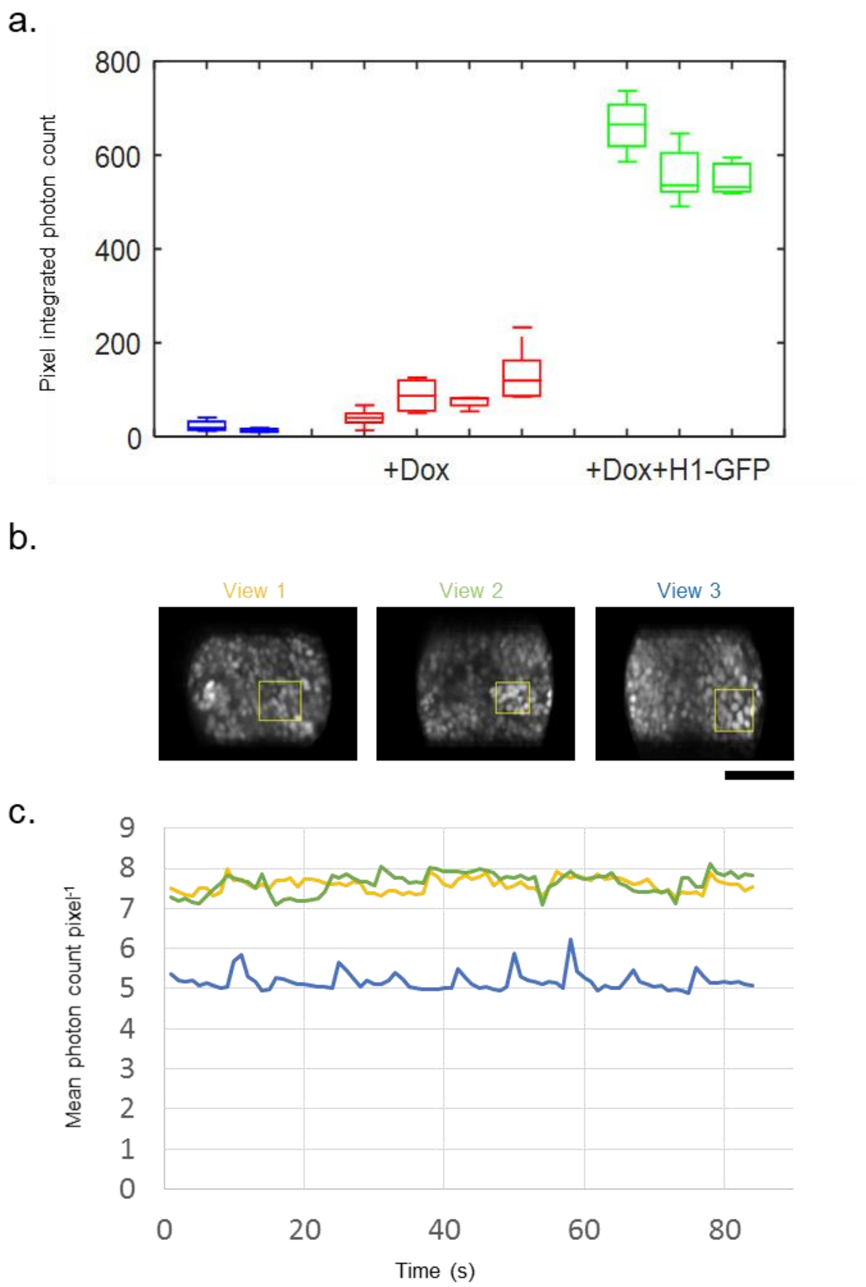

Supplementary Figure 4

Minimal direct doxorubicin excitation in vivo and lack of photobleaching

(a) Box plots show the signal intensity imaging tumors not expressing EGFP with no doxorubicin treatment i.e. tissue autofluorescence (blue boxes; n=2 mice), tumors not expressing EGFP and treated with doxorubicin at 5 mg kg^{-1} i.e. direct doxorubicin fluorescence (red boxes; n=4 mice), and tumors expressing H1-EGFP treated with doxorubicin at 5 mg kg^{-1} (green boxes; n=3 mice). On each box plot, the central mark indicates the median, and the bottom and top edges of the box indicate the interquartile range (IQR). The box plot whiskers represent either 1.5 times the IQR or the maximum/minimum data point if they are within 1.5 times the IQR. (**b)** Upper panels show three example images from different tumor nodules (scale

bar is 120 μm). (**c**) The mean photons pixel^{-1} in the indicated areas in (**b**) are plotted as a function of time, in units of seconds (s), for every frame in the acquisition with the color of the line in the chart corresponding to text labels above.

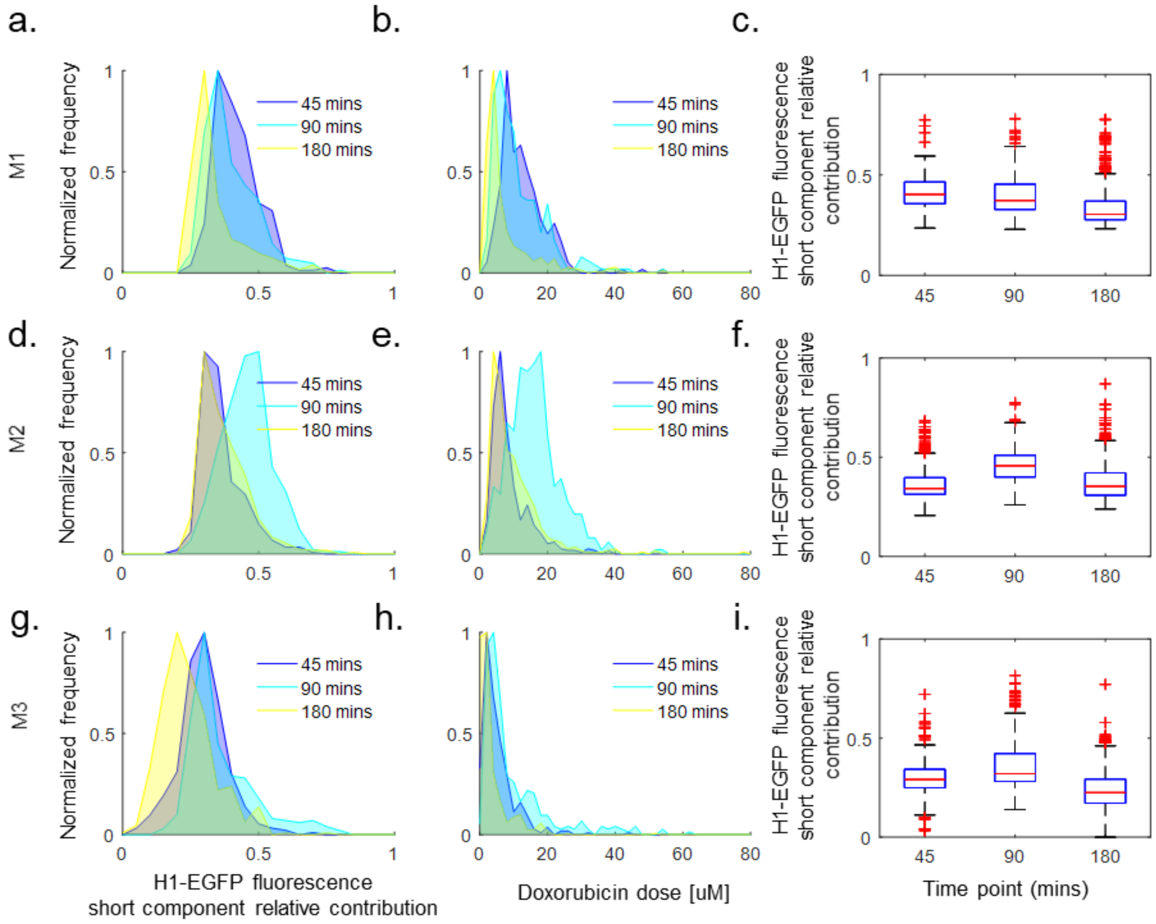

Supplementary Figure 5

Longitudinal imaging of mice at 45, 90 and 180 minutes following IP delivery of doxorubicin.

(**a**)-(**i**) show normalized histograms showing time course of distributions of the mean H1-EGFP FRETing population fraction per nucleus as above for three mice imaged longitudinally at 45, 90 and 180 minutes (mins) after intraperitoneal delivery of doxorubicin. The three sets of plots (**a**)-(**c**), (**d**)-(**f**), (**g**)-(**i**) refer to mouse 1, 2 and 3 respectively. On each box plot, the central mark indicates the median, and the bottom and top edges of the box indicate the interquartile range (IQR). The box plot whiskers represent either 1.5 times the IQR or the maximum/minimum data point if they are within 1.5 times the IQR. Outliers are shown using the red plus symbol in red and are plotted if they exceed the IQR ± whisker length.

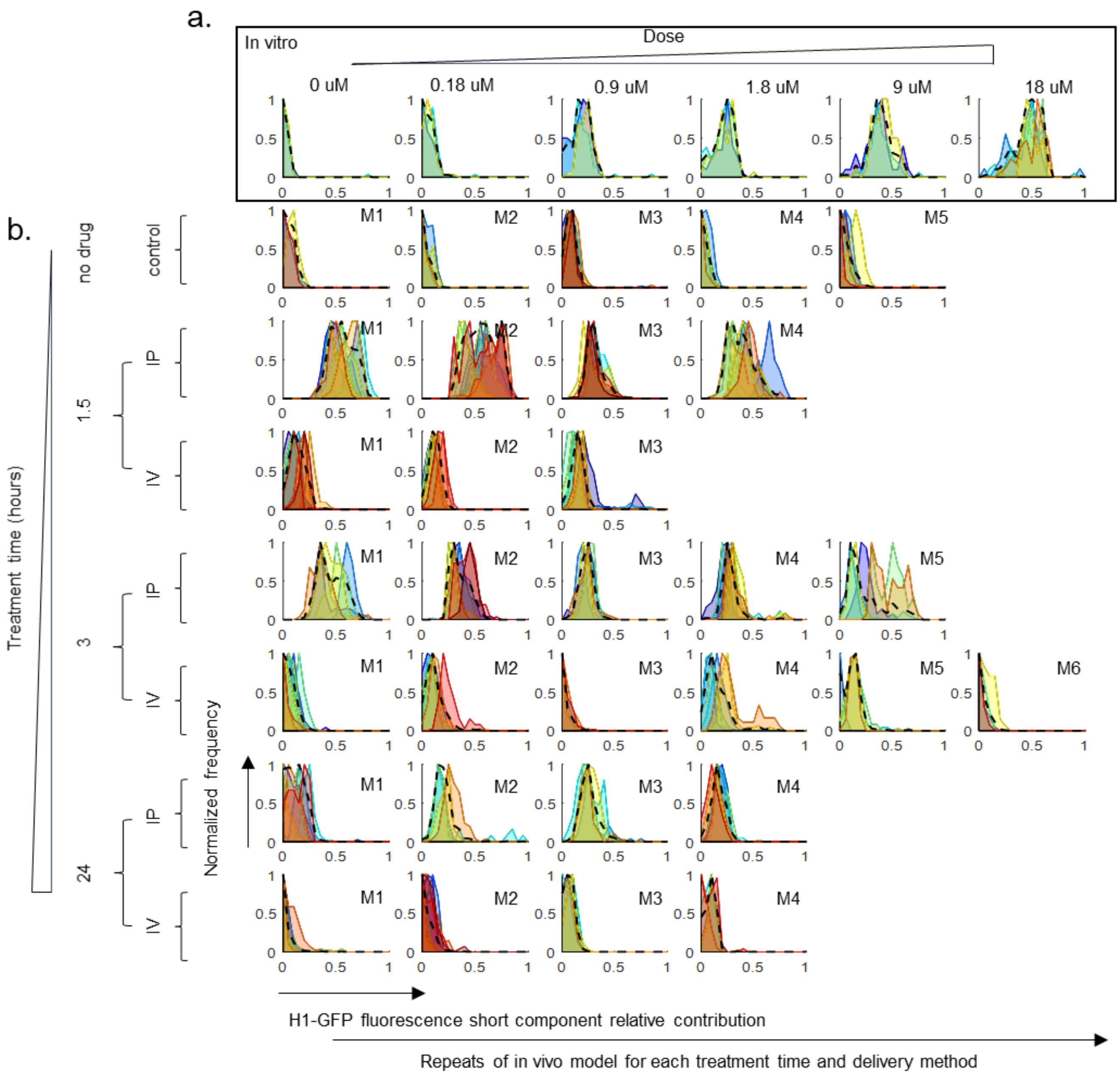

Supplementary Figure 6

H1-EGFP lifetime analysis for every field of view for 31 mice including all drug delivery routes and times

(**a**) in vitro dose response measured with the multiphoton LSM. (**b**) normalized histograms showing distributions of the mean H1-EGFP FRETing population fraction per nucleus for each mouse, each view, both drug delivery routes and all incubation times measured in vivo with the CEM. Each color-filled histogram represents a cell-wise distribution for each field of view with each sub-plot representing a different mouse (M).

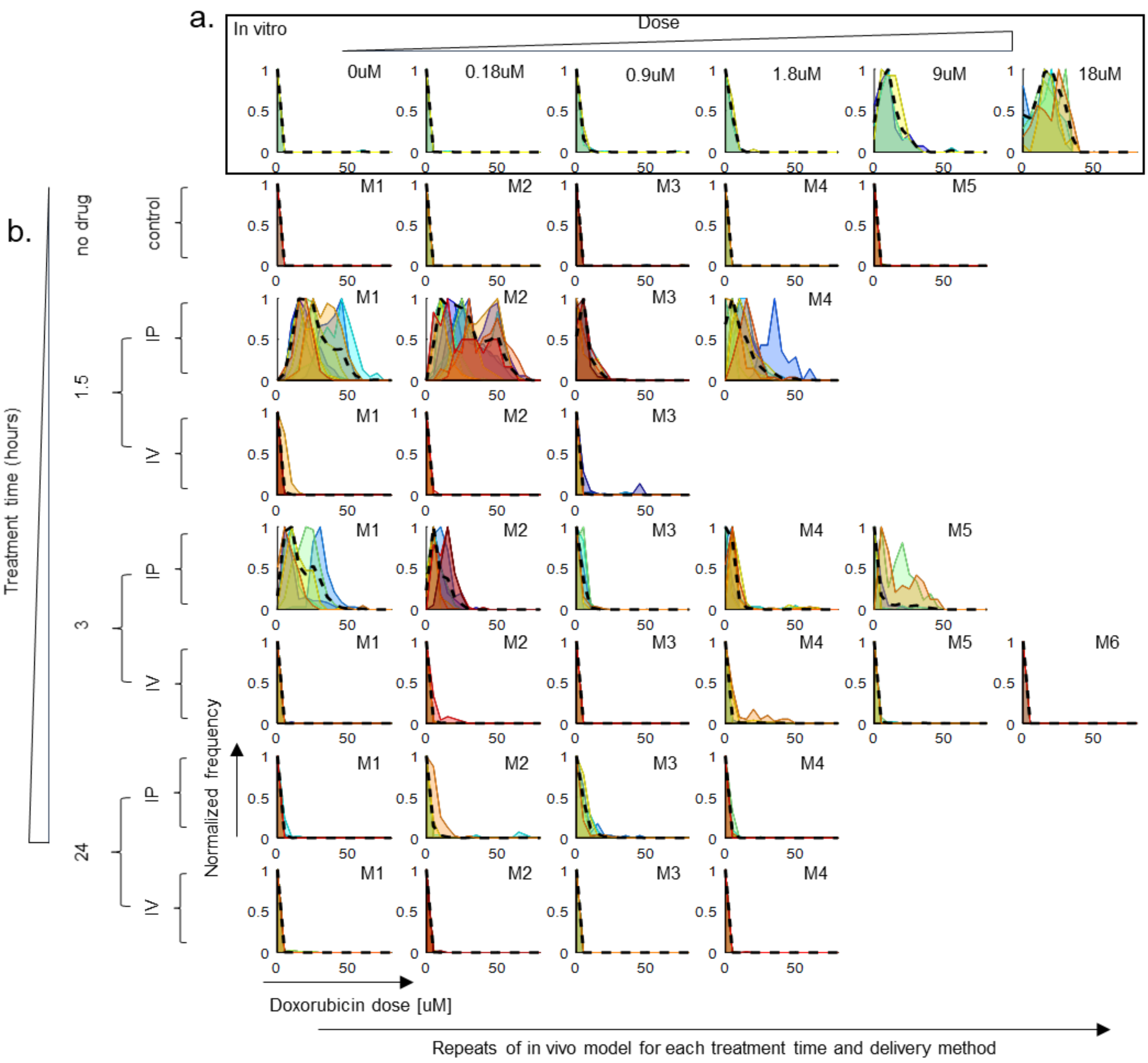

Supplementary Figure 7

Calculated doxorubicin dose for every field of view for 31 mice including all drug delivery routes and times

(**a**) in vitro dose response measured with the multiphoton LSM. (**b**) normalized histograms showing distributions calculated equivalent exposure to doxorubicin for every nucleus imaged across 31 mice, view, both drug delivery routes and all incubation times measured in vivo with the CEM. Each color-filled histogram represents a cell-wise distribution for each field of view with each subplot representing a different mouse (M). In vitro dose response measured with the multiphoton LSM is shown in the top panels.

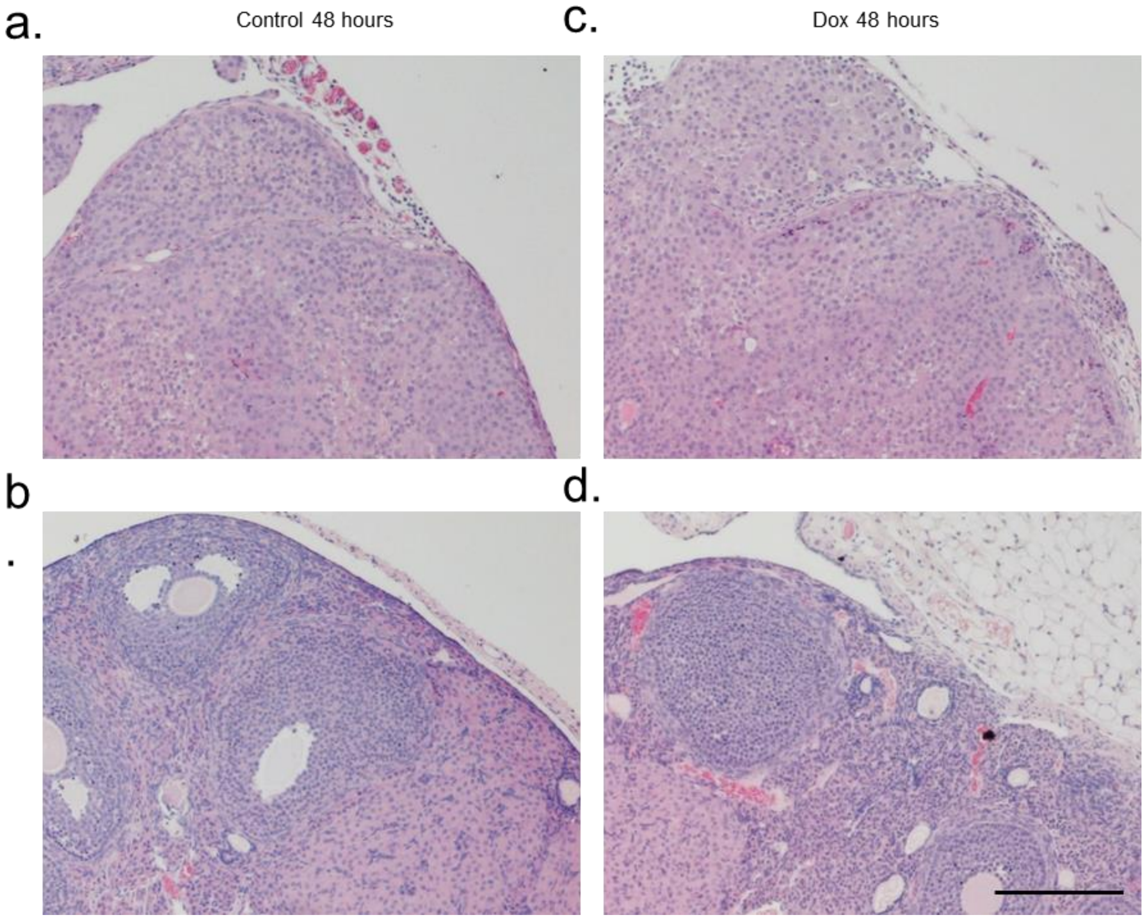

Supplementary Figure 8

Histology of control and doxorubicin treated tumors

Images show two examples of H&E stained control, (**a**), (**b**) and 5mg kg^{-1} doxorubicin treated (**c**), (**d**), IGROV-1 tumors (48hrs post doxorubicin injection). Scale bar is 200µm.

					H1-EGFP				DOX			
	Instrument	Excitation (nm)	Emission bandpass filter (nm)	Fitting method / Data used	H1-EGFP (non-FRETing) τ_1 (ps)	H1-EGFP (FRETing) τ_2 (ps)	β_1	X^2	DOX τ_1 (ps)	DOX τ_2 (ps)	DOX β_1	Dox fit X^2
In vitro	MLSM	900	465-495	Global single exponential fit / Untreated IGROV-1 H1-EGFP cells	2330	-	-	1.00	-	-	-	-
			465-495	Global double exponential fit, long fixed to 2330 ps, short fitted / Treated & untreated IGROV-1 H1-EGFP cells	-	770	0.28	1.06	-	-	-	-
	CEM	488	520-550	Global single exp fit / Untreated IGROV-1 H1-EGFP cells	2460	NA	-	1.11	1590	390	0.35	1.13
In vivo	CEM	488	520-550	Global single exp fit / Untreated IGROV-1 H1-EGFP mice	2376	NA	-	1.08	-	-	-	-

Supplementary Table 1

Measured fluorescence lifetime components

Fluorescence lifetime parameters measured for IGROV-1 cells expressing H1-GFP with emission fitted to a double exponential decay model when treated with doxorubicin when using the multiphoton laser scanning microscope (MLSM) or the confocal endomicroscope (CEM). The in vitro FLIM data was acquired from measurements of 3 or more fields of view of IGROV-1 cells in culture. The non-FRETing H1-GFP lifetime measured in vivo using the CEM is an average from measurements of 3 or more fields of view across 3 mice not treated with doxorubicin. Also included are fluorescence lifetime parameters measured using the CEM to image wild-type IGROV-1 cells treated with doxorubicin with emission fitted to a double exponential decay model.

Delivery method			Treatment time (hrs)	Nodules	Views per nodule(s)
No drug	IP	IV			
				1	4
				1	6
				1	8
				1	6
				1	7
				3	3,3,3
			1.5	2	5,4
			1.5	2	5,5
			1.5	1	8
			1.5	2	7,4
			3	1	5
			3	1	8
			3	1	8
			3	1	8
			3	1	11
			3	3	3,3,3
			3	3	3,3,3
			24	1	7
			24	1	5
			24	1	5
			24	1	7
			0.75,1.5,3	1	3,5,5
			0.75,1.5,3	1	5,6,7
			0.75,1.5,3	1	5,4,5
			1.5	2	7,7
			1.5	2	5,6
			1.5	2	5,5
			3	1	9
			3	1	7
			3	1	7
			3	1	6
			3	1	5
			3	1	4
			24	2	6,9
			24	3	4,6,5
			24	1	8
			24	1	8

Supplementary Table 2

Summary of in vivo experiments

Table summarizes all the mice imaged in this study with the drug treatment route and treatment times in hours (hrs) listed alongside the number of nodules imaged per mouse and number of fields of view imaged per nodule.

Supplementary References

1. Vogel, S. S., Nguyen, T. A., van der Meer, B. W. & Blank, P. S. The impact of heterogeneity and dark acceptor states on FRET: implications for using fluorescent protein donors and acceptors. *PLoS One* **7,** e49593 (2012).

2. Fiallo, M., Laigle, A., Borrel, M. N. & Garnier-Suillerot, A. Accumulation of degradation products of doxorubicin and pirarubicin formed in cell culture medium within sensitive and resistant cells. *Biochem. Pharmacol.* **45,** 659–65 (1993).

3. Karukstis, K. K., Thompson, E. H., Whiles, J. A. & Rosenfeld, R. J. Deciphering the fluorescence signature of daunomycin and doxorubicin. *Biophys. Chem.* **73,** 249–63 (1998).

4. Hovorka, O. *et al.* Spectral analysis of doxorubicin accumulation and the indirect quantification of its DNA intercalation. *Eur. J. Pharm. Biopharm.* **76,** 514–524 (2010).

5. Chen, N.-T. *et al.* Probing the dynamics of doxorubicin-DNA intercalation during the initial activation of apoptosis by fluorescence lifetime imaging microscopy (FLIM). *PLoS One* **7,** e44947 (2012).
