# Supplementary material for: Heterogeneity in tumor chromatin-doxorubicin binding revealed by in vivo fluorescence lifetime imaging confocal endomicroscopy
Source: Nat Commun. 2018 Jul 9;9:2662. doi: 10.1038/s41467-018-04820-6 (PMC6037736; doi:10.1038/s41467-018-04820-6)
Supplement: Supplementary file 3 — Description of Additional Supplementary Files [file 41467_2018_4820_MOESM3_ESM.pdf]

Description of Additional Supplementary Files

File Name: Supplementary Movie 1
Description: Movie showing processing of confocal endomicroscope FIFO TCSPC data. Video describing raw FIFO TCSPC frame alignment and accumulation process. FIFO TCSPC data acquired at 8.5 Hz are filtered for motion artefacts and aligned. The aligned raw frames are accumulated to make higher signal-to-noise ratio FLIM data.
